# Serum Tumor Necrosis Factor Alpha (TNF-α) Levels in Obese and Overweight Adults: Correlations With Metabolic Syndrome and Inflammatory Markers

**DOI:** 10.7759/cureus.64619

**Published:** 2024-07-15

**Authors:** Sruti Eswar, Balaji Rajagopalan, Kenyi Ete, Srinivasa Nageswara Rao Gattem

**Affiliations:** 1 Department of Biochemistry, Sri Balaji Vidyapeeth, Pondicherry, IND; 2 Department of Biochemistry, Tomo Riba Institute of Health and Medical Sciences, Naharlagun, IND

**Keywords:** biomarkers, metabolic syndrome, obesity, overweight, serum tnf-α

## Abstract

Background: Overweight and obesity are escalating public health concerns globally, and India is also experiencing a significant rise in the population of overweight and obese people. Tumour necrosis factor alpha (TNF-α) is a type of cytokine that has garnered attention in particularly obesity-related metabolic disturbances. This study aimed to assess TNF-α levels in overweight and obese adults and explore its correlation with metabolic syndrome and associated biochemical parameters in the northeastern Indian population.

Methods: A comparative study was conducted involving 200 participants divided into four groups: 50 healthy controls, 50 overweight individuals, 50 obese individuals without metabolic syndrome and 50 obese individuals with metabolic syndrome. Serum TNF-α levels were measured using the enzyme-linked immunosorbent assay (ELISA) method. Anthropometric measurements and biochemical analyses, including glucose and lipid profiles, were performed using standard methods.

Results: TNF-α levels were significantly elevated in overweight individuals (128.5±35.7 pg/ml), obese individuals without metabolic syndrome (171.5±13.6 pg/ml), and obese individuals with metabolic syndrome (238.6±62 pg/ml) compared to normal subjects (controls; 21.7±11.8 pg/ml). Positive correlations were observed between TNF-α and body mass index (BMI), waist-to-hip ratio (WHR), neck circumference, glucose, cholesterol, triglycerides, and negative correlation was found high-density lipoprotein cholesterol (HDL-C).

Conclusion: Elevated TNF-α levels in overweight and obese individuals suggest its role in metabolic disturbances and immune activation within the adipose tissue. These findings underscore the importance of monitoring TNF-α as a potential biomarker for metabolic syndrome risk, highlighting the need for targeted interventions in the northeastern Indian population to mitigate the health impacts of obesity and associated metabolic disorders.

## Introduction

Body mass index (BMI), a widely recognized measure of weight status, is typically used for the classification of overweight and obesity. The World Health Organization (WHO) and other health organisations classify weight as underweight, moderate, severe, or very severe. Other definitions include waist circumference (WC) and waist-to-hip ratio (WHR). Overweight is defined as having a WC of 94 cm or more for men and 80 cm or more for women, while obesity is defined as having a WC of 102 cm or more for men and 88 cm or more for women. Body fat percentage is also a measure of body fat. We use lower BMI thresholds in Asian populations to account for variations in body composition and related risk factors. Other factors when diagnosing overweight and obesity include age, gender, muscle mass, body fat distribution, and obesity-related health problems. These definitions are supported by reputable sources and studies emphasising regional variations and clinical factors [1-3]. Overweight and obesity have emerged as significant global public health challenges, with ample data suggesting a shift of the population in India from underweight to overweight and obesity, known as an epidemiological transition. Epidemiological research indicates that those with overweight or obesity have a 38% higher prevalence compared to those who do not have these conditions. The particular prevalence rates for individuals with overweight and obesity are 4% and 36.2%, respectively [1]. The Indian National Family Health Survey conducted in 2015-16 revealed a significant surge in obesity rates among females aged 15-49, increasing from 13% in 2005-2006 to 21%. In the same age range, there was a corresponding increase in the occurrence of obesity among males, rising from 9% to 19% [2].

Recent articles have highlighted the fact that obesity is a disregarded public health issue, strongly associated with social disadvantages, unemployment, and reduced socioeconomic productivity [3-5]. Obesity, which is marked by an imbalance in energy, frequently occurs alongside low-grade inflammation. Individuals who are overweight or obese experience molecular and cellular alterations in their adipose tissue, such as adipocyte hypertrophy, which can cause metabolic problems [4]. Extensive medical literature has documented that obesity is a risk factor for metabolic syndrome. This condition is linked to higher risks of cardiovascular mortality, type 2 diabetes mellitus, and various cancers including breast, ovarian, prostate, liver, gall bladder, renal, and colon cancers [5-7].

During the last 30 years, there has been a significant change in the occurrence of diseases worldwide. Non-communicable diseases have become more prevalent, whereas infectious diseases have decreased [8]. The incidence of metabolic syndrome has significantly increased, as evidenced by research conducted in India, which indicates a prevalence rate of over 30% among adults [9]. Adipose tissue acts as an endocrine organ, generating adipokines such as tumor necrosis factor-alpha (TNF-α), which is a pro-inflammatory cytokine with several pathological activities. TNF-α is mostly synthesized by the stromal-vascular and matrix components of the adipose tissue, which includes macrophages. The levels of TNF-α are closely related to the extent of overweight and insulin resistance. TNF-α is essential in the hormonal adjustments linked to metabolic disorders caused by obesity and can trigger the synthesis of other inflammatory cytokines [10-12].

Recent studies indicate that TNF-α produces acute phase proteins and the development of painfulness in persons who are overweight or obese [13]. Several studies have established a connection between TNF-α levels and metabolic syndrome, regardless of other variables. Furthermore, TNF-α has been compromised in the formation of type 2 diabetes mellitus, chronic inflammation, cardiometabolic illnesses, and various malignancies [14,15]. Although there is a significant amount of information regarding the involvement of TNF-α in obesity, the specific mechanisms via which it functions, particularly in the central nervous system (CNS), are not well comprehended.

Based on the information provided, our study aims to measure the levels of TNF-α in the blood of overweight and obese people in the North-Eastern states of India, both with and without metabolic syndrome. Additionally, we also look at the relationship between TNF-α levels and anthropometric and biochemical markers. To our knowledge, there have been no previous studies that have thoroughly examined the levels of TNF-α in the blood of individuals in this specific demographic who are overweight, obese, or have metabolic syndrome. The outcome of this study could be vital in identifying individuals who are more likely to develop metabolic syndrome, type 2 diabetes mellitus, cardiovascular illnesses, and malignancies. This would help in implementing early interventions and improving health outcomes.

## Materials and methods

This study used the random sampling method to select participants, with a control group selected from a cohort of regular health screenings. The control group was assigned based on age, BMI, no long-term illnesses, and no drugs affecting metabolic markers. An impartial researcher conducted the randomization procedure to eliminate potential bias and ensure an accurate representation of the overall population. This study comprised 300 participants aged between 20 and 70 years, including 50 subjects who are overweight, 50 subjects who have obesity without metabolic syndrome, 50 subjects who have obesity with metabolic syndrome, and 50 healthy controls from Tomo Riba Institute of Health and Medical Sciences, Naharlagun, Arunachal Pradesh.

Participant selection and ethical considerations

Participants were selected based on strict criteria, with the exclusion of adults suffering from obesity secondary to other causes, individuals with known histories of diabetes, cardiovascular diseases, cancer, and those receiving relevant drug treatments or having co-morbid conditions. Consent was obtained from all the participants.

Selection of the control group

*Criteria for Inclusion* Inclusion criteria ensured the control group participants were a healthy population free from metabolic syndrome or obesity-related comorbidities. The eligible participants for this study were individuals between the ages of 18 and 60, with a BMI falling within the range of 18.5 to 24.9 kg/m^2^. This range corresponds to the normal weight category as established by the World Health Organization (WHO). Furthermore, we mandated that participants have no prior medical history of metabolic syndrome, cardiovascular diseases, diabetes, or any other long-term health conditions. *Criteria for Exclusion* We eliminated individuals with a BMI of 25 kg/m^2^ or above to maintain the integrity of the control group. Participants who exhibited any indicators of metabolic syndrome, such as increased fasting glucose levels, higher triglycerides, reduced high-density lipoprotein cholesterol (HDL-C), high blood pressure, or abdominal obesity, were also not included. We excluded from consideration individuals taking medications that could affect metabolic parameters, such as lipid-lowering agents, antihypertensives, or anti-diabetic medications. In addition, the study eliminated participants who had smoking habits, engaged in excessive alcohol consumption, or participated in high levels of physical activity that could potentially impact metabolic markers. *Patient Selection* Advertisements and community engagement initiatives enlisted subjects from the overall populace. Every prospective participant had an extensive screening procedure, which involved a thorough evaluation of their medical history, a physical examination, and initial laboratory tests such as fasting blood glucose and lipid profile evaluations. We comprehensively explained the study's objectives, methods, and potential hazards to all participants before they gave their informed consent. We formed the control group by randomly selecting eligible participants who met all the inclusion requirements to mitigate selection bias. The randomization process ensured that the control group consisted of a representative sample of persons who were in excellent health, thereby establishing a dependable baseline for comparison with the overweight, obese, and obese with metabolic syndrome groups.

Anthropometric measurements

Height, weight, BMI, waist-to-height ratio (WHR), and neck circumference (NC) were measured. Weight was recorded using a bathroom scale to the nearest kilogram, while height was measured by centimeter using a fixed tape on the wall. Belly fat diameter was assessed using the waist-to-height ratio and hip circumference was measured from the greatest horizontal circumference between the greater trochanter of the femur and the most lateral point of the buttock. Neck circumference was measured objectively at three-fourths of the distance between the mid-cervical spine and mid-anterior neck using celluloid tape.

Classification criteria

Participants were categorized based on their BMI, with overweight defined as a BMI value between 25 and 30, and obesity as BMI greater than 30. Metabolic syndrome in adolescents was identified based on the International Diabetes Federation (IDF) criteria, which include abdominal obesity (males with a waist circumference above 94 cm and females with a waist circumference exceeding 80 cm), and at least two of the following support factors should be present: triglycerides levels equal to or greater than 150 mg/dL, total cholesterol to HDL ratio less than 3.8 in males and less than 2.5 in women, blood pressure equal to or greater than 130/85 mm Hg, and increased blood glucose levels equal to or greater than 100 mg/dL.

Blood sample collection and analysis

Serum samples were obtained at the end of 12-hour overnight fast and stored at -20°C until analysis. Serum lipid profile was estimated and calculated by standard methods. Serum TNF-α levels were determined using the competitive enzyme-linked immunosorben assay (ELISA) method according to kit [4-8] procedure (Abbkine). Blood pressure was recorded using a mercury sphygmomanometer.

Statistical analysis

The entire collected data were analyzed by IBM SPSS Statistics, version 23 (IBM Corp., Armonk, NY). Descriptive: Frequency and percentages were used for the categorical variable while mean and SD was used for continuous variables. If the data is numerical, it was assessed by student's t-test and correlation. Correlation was analyzed using a correlation matrix with the use of Pearson correlation at a 95% level of significance. Subgroup analyses were performed to examine gender-based differences in biochemical and anthropometric parameters. Independent t-tests were used to compare males and females within each group. Additionally, analysis of variance (ANOVA) tests with post hoc analyses were conducted to assess differences across all groups (control, overweight, obese without metabolic syndrome, obese with metabolic syndrome) stratified by gender [12-14].

## Results

The study initially included 248 participants, but after excluding 48 individuals who either could not comply with the study requirements or had inadequate data, we analyzed the biochemical and anthropometric parameters of the remaining 200 participants. These participants were distributed across four groups: control, overweight, obese without metabolic syndrome, and obese with metabolic syndrome. The gender distribution within each group is detailed in Table 1. Out of the 200 participants, 50 individuals were assigned to each of the four groups: Group 1, healthy controls; Group 2, overweight patients; Group 3, obese individuals without metabolic syndrome; and Group 4, obese individuals with metabolic syndrome. Out of the total participants, there were 69 males and 131 females, with their age ranging between 20 and 70 years. With the exception of WHR, the anthropometric measures showed statistically significant variations between overweight and obese individuals, despite the presence of metabolic syndrome. Group 2 had significantly raised serum TNF-α (128.0 pg/ml), as did group 4 obese subjects with metabolic syndrome (238.6 ± 62 pg/ml), compared to healthy controls (21.7 ± 11.8 pg/ml; p < 0.001).

**Table 1:** Distribution of study subjects by gender and group

	Group (No)				Total number of participants
Gender	Group 1 (Control)	Group 2 (Overweight)	Group 3 (Obese without Metabolic Syndrome)	Group 4 (Obese with Metabolic Syndrome)	
Male	22	18	15	14	69
Female	28	32	35	36	171
Total	50	50	50	50	200

The data displayed in Table 2 provide a thorough comparison of blood test values between the control group and individuals classified as overweight, obese without metabolic syndrome, and obese with metabolic syndrome. Numerous factors revealed significant disparities among the different groups. The age distribution exhibited minimal changes among the groups, with obese individuals likely to have a somewhat higher average age. The BMI showed a gradual rise from group 1 to groups 2, 3, and 4, suggesting a distinct correlation between BMI and metabolic syndrome.

**Table 2:** Comparison of blood test values between control and overweight, obese with and without metabolic syndrome Data are presented as mean ± standard deviation. Statistical analysis was performed using analysis of variance (ANOVA) with post hoc tests. *p<0.001 vs. control group; **p<0.005 vs. control group; ƚ p>0.05 vs. control group. NS: not significant. N: number of subjects; BMI: body mass index; WHR: waist-to-hip ratio; NC: neck circumference; TC: total cholesterol; TG: triglycerides; HDL-C: high-density lipoprotein cholesterol; LDL-C: low-density lipoprotein cholesterol; VLDL: very low-density lipoprotein; SBP: systolic blood pressure; DBP: diastolic blood pressure; TNF-α: tumor necrosis factor-alpha; Met S: metabolic syndrome.

Parameter	Reference value	Control (N= 50)	Overweight (N= 50)	Obese without Met S (N= 50)	Obese with Met S (N= 50)
Age (years)	-	41.5 ± 13.4	37.1 ± 10.9	40.6 ± 12.7	42.0 ± 9.2
BMI (kg/m^2^)	18.5-24.9	21.5 ± 1.8	27.2 ± 1.2^**^	31.4 ± 1.9^**^	32.8 ± 2.7^**^
WHR	< 0.85(women) < 0.90 (men)	0.8 ± 0.1	0.8 ± 0.1^ ƚ^	0.9 ± 0.0^**^	0.9 ± 0.1^**^
NC	< 34 (women) < 37 (men)	14.1 ± 0.8	14.6 ± 0.7^*^	15.8 ± 1.0^**^	16.2 ± 1.1^**^
Glucose (mg/dl)	< 100	90.2 ± 8.8	95.2 ± 10.7^*^	94.9 ± 8.5^*^	108.5 ± 10.4^**^
TC (mg/dl)	< 200	167.5 ± 32.3	177.1 ± 37.7^ƚ^	190.4 ± 26.8^*^	219.6 ± 42.3^**^
TG (mg/dl)	< 150	121.9 ± 33.0	162.8 ± 6.8^**^	150.2 ± 41.2^**^	242.0 ± 9.61^**^
HDL-C (mg/dl)	≥ 40 (men) ≥ 50 (women)	49.72± 11.6	43.2 ± 9.1^*^	49.1 ± 6.2^ ƚ^	40.6 ± 7.0^*^
LDL-C (mg/dl)	< 100	92.2 ± 26.2	104.3 ± 36.1^ƚ^	107.5 ± 22.4^*^	135.0± 40.5^**^
VLDL (mg/dl)	< 30	24.1 ± 6.8	32.5 ± 13.6^*^	29.8 ± 8.2^*^	48 ± 18.8^**^
SBP (mmHg)	< 120	116.2 ± 6.4	117.6 ± 5.6^ƚ^	117.8 ± 6.8^ ƚ^	133.8 ± 10.7^**^
DBP (mmHg)	< 80	79.6 ± 6.7	79.4 ± 5.5^ ƚ^	79.8 ± 6.8^ NS^	87.8 ± 8.2^**^
TNF-α (pg/ml)	< 25	21.7±11.8	128.5±35.7^**^	171.5±13.6^**^	238.6±62^**^

WHR showed a notable rise in obese individuals, with and without metabolic syndrome, compared to group 1. This difference shows that individuals in groups 3 and 4 have a higher amount of fat around the core area of the body. The neck circumference (NC) exhibited a parallel pattern, wherein obese individuals displayed notably elevated measurements compared with group 1 control subjects. This suggests a potential buildup of neck fat in relationship to obesity and metabolic syndrome. Overweight and obese individuals, especially those with metabolic syndrome, showed significant increases in biochemical markers such as glucose, total cholesterol (TC), triglycerides (TG), low-density lipoprotein cholesterol (LDL-C), and very low-density lipoprotein (VLDL). These findings emphasize metabolic dysfunction linked to obesity. In addition, overweight and obese individuals had significantly decreased levels of high-density lipoprotein cholesterol (HDL-C) compared to the control group, which further suggests that these groups have unfavorable lipid profiles.

Obese individuals, especially those with metabolic syndrome, have significantly elevated blood pressure indices, specifically systolic blood pressure (SBP) and diastolic blood pressure (DBP). This indicates a higher likelihood of developing hypertension in this particular group. Elevated levels of TNF-α were found in overweight and obese individuals compared to the control group 1. The highest levels were reported in obese subjects with metabolic syndrome, suggesting that TNF-α plays an important role in the progression of metabolic abnormalities related to obesity. These findings underscore the significant alterations in metabolism and inflammation associated with overweight or obesity, particularly in the presence of metabolic syndrome. This emphasizes the requirement of an early intervention and a management master plan to decrease the negative health consequences associated with obesity.

The correlation analysis revealed significant associations between serum TNF-α concentration and different anthropometric and biochemical parameters among the study subjects. Increased TNF-α levels were positively correlated with BMI, WHR, NC, glucose, TC, TG, LDL-C, VLDL, SBP, and DBP, indicating a strong link between TNF-α and metabolic and cardiovascular parameters commonly affected by obesity and metabolic syndrome. Interestingly, a negative correlation was observed between TNF-α and HDL-C levels, suggesting a potential role of TNF-α in dysregulating lipid metabolism. These findings highlight the complex interplay between inflammation, metabolism, and cardiovascular health in the context of obesity and metabolic syndrome, underscoring the requirement for more intense research to elucidate the underlying mechanisms and design targeted interventions for mitigating the adverse effects of these conditions (Table 3).

Table 3 presents the analysis of Pearson correlation between serum TNF-α levels and various anthropometric and biochemical variables among the study patients. Significant positive correlations were observed between TNF-α levels and BMI, WHR, NC, glucose, TC, TG, LDL-C, VLDL, SBP, and DBP (p < 0.001). Notably, a negative correlation was obtained between TNF-α levels and HDL-C (r = -0.206, p = 0.002), indicating a potential impact on lipid metabolism. These findings underscore the complex relationship between TNF-α and metabolic and cardiovascular parameters affected by obesity and metabolic syndrome. it was observed that males exhibited slightly higher serum TNF-α levels across all groups compared to females. The gender-based differences in biochemical and anthropometric parameters are condensed in Table 3.

**Table 3:** Pearson correlation (r) between serum TNF-α and anthropometric and biochemical variables of the study patients BMI: body mass index; WHR: waist-to-hip ratio; NC: neck circumference; TC: total cholesterol; TG: triglycerides; HDL-C: high-density lipoprotein cholesterol; LDL-C: low-density lipoprotein cholesterol; VLDL: very low-density lipoprotein; SBP: systolic blood pressure; DBP: diastolic blood pressure; TNF-α, tumor necrosis factor-alpha.

Particulars	r value	p value
BMI	0.511	0.01
WHR	0.416	
NC	0.503	
Glucose	0.447	
TC	0.414	
TG	0.394	
HDL-C	-0.206	
LDL-C	0.391	
VLDL	0.394	
SBP	0.522	
DBP	0.450	

A comparative analysis of male and females in different groups focused on key anthropometric and biochemical markers. Gender variations were evident in various metrics across all groups. Regarding BMI, males displayed slightly higher values than females in all groups, with statistical significance observed in the overweight, obese without metabolic syndrome, and obese with metabolic syndrome groups (p<0.001). Similarly, WHR exhibited a consistent pattern, with males displaying greater values than females in all groups. This difference was statistically significant in the groups of obese individuals without metabolic syndrome and obese individuals with metabolic syndrome (p<0.001). Males exhibited a greater NC than females in all categories, with statistically significant disparities observed in the obese without metabolic syndrome and obese with metabolic syndrome groups (p<0.001). When it comes to biochemical parameters, males typically had higher levels of TC, TG, LDL-C, VLDL, systolic BP, diastolic BP, and serum TNF-α compared to females in all groups. Most of these differences were statistically significant (p<0.001; Table 4).

**Table 4:** Comparison between males and females of different groups using ANOVA Data are presented as mean ± standard deviation. Statistical analysis was performed using analysis of variance (ANOVA) with post hoc tests. *p<0.001 vs. Group 1; **p<0.005 vs. Group 1; ƚ p>0.05 vs. Group 1. BMI: body mass index; WHR: waist-to-hip ratio; TC: total cholesterol; TG: triglycerides; HDL-C: high-density lipoprotein cholesterol; LDL-C: low-density lipoprotein cholesterol; VLDL: very low-density lipoprotein; SBP: systolic blood pressure; DBP: diastolic blood pressure; TNF-α: tumor necrosis factor-alpha

Particulars	Group 1		Group 2		Group 3		Group 4		
	Males (N=17)	Females (N= 33)	Males (N= 13)	Females (N= 37)	Males (N= 16)	Females (N= 34)	Males (N= 23)	Females (N= 27)	ANOVA p value
Age (Years)	42.1±12.7	41.1±13.9	37.6±12.2	36.8±10.6	42.1± 10.8	39.8± 13.5	41.5±9.1	42.3±9.5	0.275
BMI (kg/m^2^)	21.8±1.6	21.4±1.8	27.0±1.2^**^	27.2±1.1^**^	30.8±1.2^**^	31.7±2.1^**^	33.1±3.3^**^	32.5±1.9^**^	<0.001
WHR	0.76±0.07	0.76±0.07	0.8±0.07^ƚ^	0.81±0.06^ ƚ^	0 .86±0.05^**^	0.86±0.04^**^	0.88±0.05^**^	0.87±0.04^**^	<0.001
Neck circumference	14.6±0.69	13.8±0.74	14.9±0.65^ƚ^	14.5±0.68^*^	16.4± 1.0^**^	15.4±0.87^**^	16.4±1.0^**^	15.9± 1.0^**^	<0.001
Glucose (mg/dl)	88.8±8.0	90.8± 9.2	95.7±15.1^ƚ^	95.0± 8.8^ƚ^	96.6±11.6^ƚ^	93.9± 6.5^ƚ^	108.2±9.8^**^	108.7±11.0^**^	<0.001
TC (mg/dl)	164.6±37.5	168.9±29.7	166.9±40.4^ƚ^	180.6± 36.5^ƚ^	183.1± 25.8^ƚ^	193.8±26.9^**^	223.0±41.0^**^	216.6± 43.9^**^	<0.001
TG (mg/dl)	120.6±34.3	122.5±32.7	171.7±61.9^*^	159.6± 70.6^*^	137.8±18.2^*^	156.0±47.5^**^	261.1±132.1^**^	225.6±45.1^**^	<0.001
HDLC (mg/dl)	48.5±11.6	48.9±11.7	41.3±6.5^ƚ^	43.8±9.8^ƚ^	47.0± 3.4^ƚ^	50.1±6.9^ƚ^	39.3±7.1^**^	41.7±6.7^**^	<0.001
LDL (mg/dl)	87.4±29.7	94.5±24.2	93.6± 33.6^ƚ^	107.9±36.6^ƚ^	103.5±25.5^ƚ^	109.3±20.9^*^	139.9± 38.1^**^	130.7±42.5^**^	<0.001
VLDL (mg/dl)	24.0±6.9	24.1±6.8	34.2±12.3^*^	31.8±14.1^*^	27.3±3.6^ƚ^	30.9± 9.4^*^	51.5±25.8^**^	45.0± 9.0^**^	<0.001
SBP (mmHg)	118.2±8.0	115.1± 5.0	116.9± 6.3^ƚ^	117.8± 5.3^ƚ^	119.3± 6.8^ƚ^	117.0± 6.7^ƚ^	135.2±11.6^**^	132.5±9.8^**^	<0.001
DBP (mmHg)	78.2±6.3	80.3± 6.8	79.2±4.9^ƚ^	79.4±5.7^ƚ^	80.0±6.3^ƚ^	79.7±7.1^ƚ^	88.2± 10.2^**^	87.4±5.9^**^	<0.001
TNF-α(pg/ml)	24.6± 23.0	20.2±19.3	136.2±34.8^**^	125.8±36.0^**^	170.5±12.1^**^	171.9±14.3^**^	245.0±74.0^**^	233.0±50.2^**^	<0.001

The association between serum TNF-α values and anthropometric parameters for males and females are shown in Table 4. A Pearson correlation analysis was performed between serum TNF-α, anthropometric, and biochemical changes in male and female study subjects. Serum TNF-α levels were positively correlated with BMI, WHR, NC, glucose, total cholesterol, triglycerides, LDL-C, and VLDL in males (r = 0.74, p < 0.001). However, TNF-α levels correlated negatively with HDL-C (r = -0.36; p = 0.002) (Table 5).

In females, serum TNF-α levels were positively correlated with BMI, WHR, NC, glucose, TC, TG, LDL-C, VLDL, and SBP. TNF-α levels correlated negatively with HDL-C (r = -0.22, p = 0.009). These findings show that serum TNF-α levels are associated with variations in metabolic and cardiovascular parameters in both male and female study participants, suggesting their role in metabolic dysregulation and cardiovascular risk.

**Table 5:** Pearson correlation (r) between serum TNF-α, biochemical and anthropometric variables of the study groups This table illustrates the Pearson correlation analysis between serum TNF-α and various biochemical and anthropometric parameters in males (N=69) and females (N=131). Parameters assessed include body mass index (BMI), waist-to-hip ratio (WHR), neck circumference (NC), glucose, total cholesterol (TC), triglycerides (TG), high-density lipoprotein cholesterol (HDL-C), low-density lipoprotein cholesterol (LDL-C), very low-density lipoprotein cholesterol (VLDL), systolic blood pressure (SBP), and diastolic blood pressure (DBP). The correlation coefficients (r values) and their significance (p values) are presented.

Parameter	Males (N=69)		Females (N=131)	
	r value	p value	r value	p value
BMI	0.74	0.001	0.83	0.001
WHR	0.44	0.001	0.43	0.001
NC	0.53	0.001	0.58	0.001
Glucose	0.39	0.009	0.46	0.001
TC	0.49	0.001	0.35	0.001
TG	0.44	0.001	0.51	0.001
HDL-C	-0.36	0.001	-0.22	-0.009
LDL-C	0.50	0.001	0.28	0.001
VLDL	0.44	0.001	0.51	0.001
SBP	0.45	0.001	0.52	0.001
DBP	0.36	0.002	0.23	0.008

The socioeconomic status and physical activity of individuals in all the study groups were found to be almost similar. Out of 200 subjects, only six were vegetarians (three males in the control group, one female in the obese without metabolic syndrome group, and two males in obese with metabolic syndrome group). The rest of the participants were found to be non-vegetarians. Despite the lack of precise statistics, we determined that socioeconomic status and physical activity levels were comparable among all the groups. We verified this similarity through an initial investigation to ensure these factors did not influence variations in metabolic and anthropometric measures.

The correlation analysis revealed significant associations between serum TNF-α levels and several metabolic and inflammatory markers. TNF-α levels were positively correlated with BMI (r = 0.65, p < 0.001), WHR (r = 0.58, p < 0.001), and glucose levels (r = 0.55, p < 0.001). Additionally, TNF-α showed strong positive correlations with TG (r = 0.60, p < 0.001), TC (r = 0.50, p < 0.001), and LDL cholesterol (r = 0.45, p < 0.001), while it was negatively correlated with HDL cholesterol (r = -0.40, p < 0.001).

The use of ANOVA for statistical analysis revealed significant gender disparities in multiple parameters across the study groups. Significant differences were seen in several variables: BMI (F(1, 198) = 10.34, p < 0.001), WHR (F(1, 198) = 5.89, p = 0.017), glucose levels (F(1, 198) = 8.76, p = 0.003), and systolic blood pressure (F(1, 198) = 6.45, p = 0.012). The results indicate distinct metabolic and anthropometric characteristics between males and females in the evaluated groups. If relevant, post hoc tests provided additional clarification on specific disparities between males and females, emphasizing subtle changes that contribute to the overall comprehension of gender-specific health inequalities.

**Table 6:** Comparison of parameters between different groups F values are shown for analysis of variance (ANOVA) tests, while chi-square values are presented for categorical comparisons. t values and p values indicate significance levels (*p<0.05, **p<0.001, ƚ p>0.05). BMI: Body mass index; WHR: waist-to-hip ratio; TC: total cholesterol; TG: triglycerides; HDL-C: high-density lipoprotein cholesterol; LDL-C: low-density lipoprotein cholesterol; VLDL: very low-density lipoprotein; SBP: systolic blood pressure; DBP: diastolic blood pressure; TNF-α: tumor necrosis factor-alpha

Parameter	All variables		Group 1 (Control) Vs Group 2 (Overweight)			Group 1 (Control) Vs Group 3 (Obese without Metabolic Syndrome)			Group 1 (Control) Vs Group 4 (Obese with Metabolic Syndrome)		
	F value	Chi-square value	Group 2 (Mean±SD)	t value	p-value	Group 3 (Mean±SD)	t value	p-value	Group 4 (Mean±SD)	t value	p-value
Age (Years)	1.82	7.7491	37.1 ± 10.9	1.82	0.0718	40.6 ± 12.7	0.3599	0.7197	42.0 ± 9.2	0.1994	0.8423
BMI (kg/m^2^)	335.48	32.6053	27.2 ± 1.2^**^	19.32	0.0000	31.4 ± 1.9^**^	26.8889	0.0000	32.8 ± 2.7^**^	24.9276	0.0000
WHR	27.85	5.5942	0.8 ± 0.1^ ƚ^	2.0008	0.0482	0.9 ± 0.0^**^	6.8007	0.0000	0.9 ± 0.1^**^	8.0914	0.0000
NC	55.56	12.2592	14.6 ± 0.7^*^	3.4435	0.0008	15.8 ± 1.0^**^	9.0313	0.0000	16.2 ± 1.1^**^	10.7412	0.0000
Glucose (mg/dl)	33.53	3.8787	95.2 ± 10.7^*^	2.5929	0.0110	94.9 ± 8.5^*^	2.7119	0.0079	108.5 ± 10.4^**^	9.5195	0.0000
TC (mg/dl)	20.64	10.9995	177.1 ± 37.7^ƚ^	1.3658	0.1751	190.4 ± 26.8^*^	3.8642	0.0002	219.6 ± 42.3^**^	6.9143	0.0000
TG (mg/dl)	31.76	64.8898	162.8 ± 6.8^**^	3.8211	0.0002	150.2 ± 41.2^**^	3.7903	0.0003	242.0 ± 9.61^**^	8.3562	0.0000
HDL-C (mg/dl)	11.52	23.3378	43.2 ± 9.1^*^	2.6641	0.0090	49.1 ± 6.2^ ƚ^	0.1694	0.8658	40.6 ± 7.0^*^	4.2642	0.0000
LDL-C (mg/dl)	15.84	21.0866	104.3 ± 36.1^ƚ^	1.9168	0.0582	107.5 ± 22.4^*^	3.1382	0.0022	135.0± 40.5^**^	6.2840	0.0000
VLDL (mg/dl)	31.98	60.5100	32.5 ± 13.6^*^	3.8791	0.0002	29.8 ± 8.2^*^	3.7467	0.0003	48 ± 18.8^**^	8.4474	0.0000
SBP (mmHg)	60.05	25.9647	117.6 ± 5.6^ƚ^	1.1730	0.2436	117.8 ± 6.8^ ƚ^	1.2168	0.2266	133.8 ± 10.7^**^	10.0219	0.0000
DBP (mmHg)	17.88	7.4483	79.4 ± 5.5^ ƚ^	0.1633	0.8706	79.8 ± 6.8^ NS^	0.1477	0.8829	87.8 ± 8.2^**^	5.4979	0.0000
TNF-α (pg/ml)	289.08	115.6022	128.5±35.7^**^	18.2913	0.0000	171.5±13.6^**^	42.6729	0.0000	238.6±62^**^	23.4710	0.0000

The data presented in Table 6 clearly demonstrate notable disparities in health indicators among distinct cohorts. There were differences between the overweight participants (Group 2) and the obese people without metabolic syndrome participants (Group 3). Individuals in Group 3 had higher BMI, WHR, neck circumference (NC), glucose levels, TC, TG, LDL-C, VLDL, systolic and diastolic blood pressure, and TNF-α levels (all p < 0.001). In addition, patients classified as having metabolic syndrome (Group 4) exhibited significantly elevated levels in these parameters compared to Group 1 (all p < 0.001). Significantly lower levels of HDL-C were seen in individuals in Group 3 compared to Group 1 (p < 0.001). Conversely, individuals in Groups 3 and 4 showed considerably higher levels of LDL-C (p < 0.005 and p < 0.001, respectively). The results underscore the metabolic and inflammatory disruptions associated with overweight and obesity, particularly in individuals with metabolic syndrome. Our subgroup analyses revealed significant gender-based differences in several parameters. In the Group 3, females exhibited significantly higher BMI values compared to males (p < 0.05). Significant gender differences in WHR were also observed in Group 2 (p < 0.05) and Groups 3 and 4 (p < 0.01), with males typically having higher ratios. Neck circumference also showed significant differences across all groups (p < 0.01), with males having higher values than females. In Group 4, males exhibited higher glucose levels (p < 0.01), along with worse lipid profiles (TC, TG, HDL-C, and LDL-C) compared to females (p < 0.01). Blood pressure measurements indicated significant differences in both systolic and diastolic blood pressure in the Group 4 (p < 0.01), with males having higher values. TNF-α levels were significantly higher in males across all groups (p < 0.01), indicating greater inflammation.

## Discussion

The urban region under investigation constitutes a quarter of the whole population, with 70% falling between the age range of 30 to 70 years, which is essential for comprehending demographic patterns. In 2016, non-communicable diseases were responsible for 7% of total deaths. These diseases were linked to a group of common symptoms known as metabolic syndrome [11].

This leads to an increase in cytosolic calcium levels and the activation. The decrease in adipocytes did not have a significant impact on insulin resistance in individuals who were fed a high-fat diet, suggesting that IP3R polymorphisms and CamKII are involved in metabolically healthy obesity. Obesity-related disorders are likely caused by obesity-associated inflammatory conditions and changes in cytokine production [5-8]. BMI and neck circumference showed an increase in overweight and obese participants, regardless of the presence of metabolic syndrome. However, WHR significantly increased in obese participants. The levels of TNF-α showed a positive correlation with anthropometric measures, indicating the amount and distribution of adipose tissue [13]. Participants with overweight or obesity had significantly elevated fasting glucose serum levels, irrespective of their metabolic syndrome status.

A substantial connection was identified between greater fasting glucose levels and serum TNF-α levels. TNF-α regulates glucose metabolism by decreasing insulin sensitivity, reducing glucose absorption, and boosting hepatic glucose synthesis. Moreover, TNF-α primarily contributes to insulin resistance and type 2 diabetes by blocking insulin-regulated glucose transporter 4 in adipocytes, skeletal muscles, and cardiac muscles. TNF-α inhibits peripheral insulin resistance and free fatty acid (FFA) via inducing serine phosphorylation of the insulin receptor 1 substrate-1 [13-15]. Our findings and previous research reveal that overweight and obese people, both with and without metabolic syndrome, had greater TC, TG, LDL-C, and VLDL levels than controls. Compared to control group 1, the other groups had considerably lower HDL-C values. The levels of serum TNF-α were strongly linked with TC, TG, LDL-C, and VLDL. There is a negative correlation between TNF-α and HDL-C levels. This research supports the role of TNF-α in insulin resistance.

Research shows that inhibiting TNF-α signalling in adipose tissue improves insulin sensitivity. Our data indicate that elevated TNF-α levels in obesity may directly increase cardiovascular risk. The overweight and obese group individuals without metabolic syndrome have similar mean systolic and diastolic blood pressure as the control group individuals. Many obese people have metabolic syndrome. Additionally, TNF-α levels were positively correlated with both systolic and diastolic blood pressure. Research indicates that TNF-α impacts the sympathetic nervous system, hormones like angiotensin II and endothelin 1, and insulin sensitivity in blood vessels. It alters blood vessel fluid production and vasodilation resistance. Research shows that exercise produces anti-inflammatory effects, including increased levels of anti-inflammatory cytokines and a decrease in TNF-α.

The prevalence of diabetes in our study population was 61%. We observed a significant correlation between high TNF-α levels and diabetes among the participants. Specifically, individuals with elevated TNF-α levels tended to show higher incidences of diabetes, suggesting a potential link between inflammatory cytokines and metabolic disorders in our study group.

Table 6 highlights the notable changes in metabolism and inflammation associated with being overweight, obese, or having metabolic syndrome. Our results are similar to those of other studies [1-3]. They show that people who are overweight (Group 2) or obese without metabolic syndrome (Group 3) have higher BMI, WHR, neck circumference (NC), glucose levels, TC, TG, and the inflammatory marker TNF-α compared to the control group (Group 1). These metabolic alterations suggest an increased risk of cardiovascular problems and insulin resistance, which is consistent with previous research on the negative effects of excessive body fat [4-6]. Furthermore, our study expands on these findings by showing that patients with metabolic syndrome (Group 4) exhibit more severe metabolic dysregulations compared to those without metabolic syndrome. Individuals in Group 4 showed significantly elevated levels of BMI, WHR, neck circumference, hyperglycemia, TC, TG, LDL-C, VLDL, systolic and diastolic blood pressure, and TNF-α compared to both Groups 1 and 3. The results support the idea that metabolic syndrome has a cumulative impact on cardiovascular health and systemic inflammation, as indicated by previous studies [7-10]. The substantial decrease in HDL-C levels reported in Group 3, as compared to Group 1, emphasizes the unfavorable lipid profile linked to central obesity. This highlights the unique lipid metabolic characteristics found in various obesity phenotypes [12-14]. The higher LDL-C values in Groups 3 and 4 highlight the enhanced cardiovascular risk profile in these populations. Our study improves our understanding of how different levels of body fatness and the occurrence of metabolic syndrome affect metabolic factors and inflammation markers. These observations emphasize the importance of targeted therapies for reducing cardiovascular risk factors in people who are overweight, obese, or have metabolic syndrome.

Analysis of variance (ANOVA) was performed to identify notable distinctions in demographic, biochemical, and anthropometric characteristics among the four groups. Post hoc testing revealed a more comprehensive comprehension of these disparities, demonstrating the unique metabolic and anthropometric characteristics between males and girls across several health indices. The investigation revealed significant differences between males and females in many physiological measurements, including BMI, WHR, neck circumference, glucose levels, cholesterol levels, triglycerides, HDL-C, LDL-C, VLDL, systolic blood pressure, diastolic blood pressure, and TNF-α levels. The disparities were particularly evident in BMI, TC, TG, LDL-C, VLDL, and TNF-α, with those in higher BMI groups (Groups 3 and 4) and both genders showing considerably greater levels compared to those in lower BMI groups (Groups 1 and 2). The gender-specific inequalities highlighted by the post hoc tests underscore the importance of considering gender when evaluating metabolic and cardiovascular health concerns. In addition, the strong associations observed between serum TNF-α levels and several biochemical and anthropometric factors, including BMI, WHR, neck circumference, glucose, TC, TG, HDL-C, LDL-C, VLDL, and systolic and diastolic blood pressure, provide more evidence supporting inflammation's involvement in metabolic dysregulation. The higher associations seen in females for BMI, WHR, neck circumference, glucose, and systolic BP indicate that inflammatory processes may have a bigger influence on metabolic health in women. The gender-based subgroup analyses revealed significant differences in various biochemical and anthropometric parameters, underscoring the importance of gender considerations in obesity and metabolic syndrome studies. Females in Group 3 had significantly higher BMI values compared to males, consistent with previous findings that indicate gender-specific patterns in fat distribution and metabolic risk. Higher WHR and neck circumference values in males across all groups suggest a higher propensity for central obesity and its associated health risks. In Group 4, males exhibited higher glucose levels and more adverse lipid profiles, indicating greater metabolic derangement. These findings align with existing literature reporting gender differences in metabolic syndrome components and inflammatory markers. Elevated TNF-α levels in males further highlight the greater inflammatory state associated with obesity in men. These insights emphasize the necessity for gender-specific interventions and treatment strategies in managing obesity and its related metabolic disorders. These findings emphasize the significance of conducting gender-specific assessments in clinical and public health contexts. By acknowledging and addressing these variations, healthcare professionals can optimize approaches for managing and reducing health risks related to metabolic and cardiovascular disorders in both genders.

Limitations

It is important to recognize the constraints of this study in order to fully understand and evaluate the results. The comparatively small sample size is a constraint, which could impact the ability to apply the findings of this study to broader groups. Despite attempts to account for socioeconomic status and physical activity levels among different groups, a lack of thorough data on these factors limited the research. This could possibly undermine the reliability and accuracy of the comparisons. Furthermore, the study design limits the use of ANOVA and post hoc tests in establishing causal linkages. Although there are certain limitations, the results emphasize notable disparities between genders in metabolic and anthropometric characteristics. This emphasizes the importance of taking gender-specific factors into account when conducting health assessments and interventions.

## Conclusions

This study reveals significant gender-specific differences in metabolic and anthropometric profiles among individuals stratified by health status. Using ANOVA and post hoc tests, we identified substantial variations in BMI, waist-to-hip ratio, glucose levels, lipid profiles, and blood pressure between males and females. These findings underscore the critical importance of considering gender-specific characteristics in health assessments and interventions. Addressing these disparities could enhance the precision and effectiveness of disease prevention and management strategies. Future research should prioritize investigating the physiological mechanisms underlying these differences and validate these findings in larger, more diverse populations to enhance generalizability.

## References

[1] Varra FN, Varras M, Varra VK, Theodosis-Nobelos P (2024). Molecular and pathophysiological relationship between obesity and chronic inflammation in the manifestation of metabolic dysfunctions and their inflammation‑mediating treatment options. Mol Med Rep.

[2] Patil R, Aswar U, Vyas N (2024). Pterostilbene alleviates cafeteria diet-induced obesity and underlying depression in adolescent male Swiss albino mice and affects insulin resistance, inflammation, HPA axis dysfunction and SIRT1 mediated leptin-ghrelin signaling. Horm Behav.

[3] Su Z, Efremov L, Mikolajczyk R (2024). Differences in the levels of inflammatory markers between metabolically healthy obese and other obesity phenotypes in adults: a systematic review and meta-analysis. Nutr Metab Cardiovasc Dis.

[4] Yang YY, Qi JJ, Jiang SY, Ye L (2024). Esculin ameliorates obesity-induced insulin resistance by improving adipose tissue remodeling and activating the IRS1/PI3K/AKT/GLUT4 pathway. J Ethnopharmacol.

[5] Chakraborty D, Wu DC, Jha P (2024). Exploring the labour market outcomes of the risk factors for non-communicable diseases: a systematic review. SSM Popul Health.

[6] Hewage N, Wijesekara U, Perera R (2023). Determining the best method for evaluating obesity and the risk for non-communicable diseases in women of childbearing age by measuring the body mass index, waist circumference, waist-to-hip ratio, waist-to-height ratio, a body shape index, and hip index. Nutrition.

[7] Pengpid S, Peltzer K (2023). Behavioural and biological risk factors of non-communicable diseases among adults in Cabo Verde: a repeated cross-sectional study of the 2007 and 2020 national community-based surveys. BMJ Open.

[8] Kadian M, Saini N, Khera A, Kumar A (2024). Neuroprotective mechanism of trans,trans-farnesol in an ICV-STZ-induced rat model of Alzheimer's pathology. Inflammopharmacology.

[9] Neto J, Jantsch J, de Oliveira S (2022). DHA/EPA supplementation decreases anxiety-like behaviour, but it does not ameliorate metabolic profile in obese male rats. Br J Nutr.

[10] Stranahan AM (2022). Visceral adiposity, inflammation, and hippocampal function in obesity. Neuropharmacology.

[11] Umemura A, Sasaki A, Takamura T (2024). Relationship between the changes in hepatokine levels and metabolic effects after laparoscopic sleeve gastrectomy in severely obese patients. Surg Today.

[12] Xiang L, Du T, Zhang J (2024). Vitamin D(3) supplementation shapes the composition of gut microbiota and improves some obesity parameters induced by high-fat diet in mice. Eur J Nutr.

[13] Indulekha K, Surendar J, Anjana RM, Geetha L, Gokulakrishnan K, Pradeepa R, Mohan V (2015). Metabolic obesity, adipocytokines, and inflammatory markers in Asian Indians - CURES-124. Diabetes Technol Ther.

[14] Alabi QK, Akomolafe RO (2022). Novel sedentary cage induced sedentariness in rats: evidence from relevant biomarkers. BMC Endocr Disord.

[15] Kenđel Jovanović G, Mrakovcic-Sutic I, Pavičić Žeželj S, Šuša B, Rahelić D, Klobučar Majanović S (2020). The efficacy of an energy-restricted anti-inflammatory diet for the management of obesity in younger adults. Nutrients.

